# Synthesis of Novel Glycerol-Derived 1,2,3-Triazoles and Evaluation of Their Fungicide, Phytotoxic and Cytotoxic Activities

**DOI:** 10.3390/molecules22101666

**Published:** 2017-10-07

**Authors:** Adilson Vidal Costa, Marcos Vinicius Lacerda de Oliveira, Roberta Tristão Pinto, Luiza Carvalheira Moreira, Ediellen Mayara Corrêa Gomes, Thammyres de Assis Alves, Patrícia Fontes Pinheiro, Vagner Tebaldi de Queiroz, Larissa Fonseca Andrade Vieira, Robson Ricardo Teixeira, Waldir Cintra de Jesus Júnior

**Affiliations:** 1 Graduate Program in Agrochemistry, Universidade Federal do Espírito Santo, Alto Universitário, S/N, Guararema, Alegre ES 29500-000, Brazil; marcao_lacerda@hotmail.com (M.V.L.O.); roberta_tristao@hotmail.com (R.T.P.); luiza.carvalheira@yahoo.com.br (L.C.M.); patriciafontespinheiro@yahoo.com.br (P.F.P.); vagnertq@gmail.com (V.T.Q.); 2 Graduate Program in Plant Production, Universidade Federal do Espírito Santo, Alto Universitário, S/N, Guararema, Alegre ES 29500-000, Brazil; ediellengomes@hotmail.com; 3 Graduate Program in Biotechnology, Universidade Federal do Espírito Santo, Alto Universitário, S/N, Guararema, Alegre ES 29500-000, Brazil; thammyresalves@gmail.com; 4 Department of Biology, Universidade Federal de Lavras, Lavras MG 37200-000, Brazil; lfandrade.vieira@gmail.com; 5 Departament of Chemistry, Universidade Federal de Viçosa, Av. P.H. Rolfs, S/N, Viçosa MG 36570-900, Brazil; robsonr.teixeira@ufv.br; 6 Universidade Federal de São Carlos, Campus Lagoa do Sino, Buri SP 18290-000, Brazil; wcintra@yahoo.com

**Keywords:** glycerol, 1,2,3-triazoles, fungicide, cytotoxic activity, click chemistry

## Abstract

The synthesis of a series of 1,2,3-triazoles using glycerol as starting material is described. The key step in the preparation of these triazolic derivatives is the copper(I)-catalyzed azide-alkyne cycloaddition (CuAAC), also known as click reaction, between 4-(azidomethyl)-2,2-dimethyl-1,3-dioxolane (**3**) and different terminal alkynes. The eight prepared derivatives were evaluated with regard to their fungicide, phytotoxic and cytotoxic activities. The fungicidal activity was assessed in vitro against *Colletotrichum gloeosporioides*, the causative agent of papaya anthracnose. It was found that the compounds 1-(1-((2,2-dimethyl-1,3-dioxolan-4-yl)methyl)-1*H*-1,2,3-triazol-4-yl)-cyclo-hexanol (**4g**) and 2-(1-((2,2-dimethyl-1,3-dioxolan-4-yl)methyl)-1*H*-1,2,3-triazol-4-yl)propan-2-ol (**4h**) demonstrated high efficiency in controlling *C. gloeosporioides* when compared to the commercial fungicide tebuconazole. The triazoles did not present any phytotoxic effect when evaluated against *Lactuca sativa*. However, five derivatives were mitodepressive, inducing cell death detected by the presence of condensed nuclei and acted as aneugenic agents in the cell cycle of *L. sativa*. It is believed that glycerol derivatives bearing 1,2,3-triazole functionalities may represent a promising scaffold to be explored for the development of new agents to control *C. gloeosporioides*.

## 1. Introduction

The 1,2,3-triazoles are five-membered nitrogenated aromatic heterocyclic molecules of exclusively synthetic origin, remarkably stable and essentially inert to oxidation, reduction and hydrolysis [1]. This class of compounds has attracted the attention of researchers because of its high effectiveness, low toxicity and vast range of biological activities, including antibacterial [2], cytotoxicity [3], antitumor [4], antiprotozoal [5], antifungal [6,7], antimalarial [8], tripanossomicide [9], and phytotoxicity effects [10], among others.

Among the available methodologies to prepare triazoles, the copper(I)-catalyzed azide-alkyne cycloaddition (CuAAC), also known as click reaction, proposed by Sharpless and Meldal, stands out as a versatile strategy affording products in high yields and being extremely useful in the preparation of 1,4-disubstituted 1,2,3-triazoles [11].

Glycerol is a natural polyol discovered in 1779 by the Swedish scientist Carl Wilhelm Scheele [12]. Its first industrial application is related to the transformation of glycerol to nitroglycerin as reported in 1860 by Alfred Nobel [13]. Since then, chemists started to find applications for glycerol, which it is widely used in the pharmaceutical and cosmetic industries. Other applications include its uses as a plasticizer in the polymer industry and as a moistening agent or solvent in the food industry [14]. This compound has also found several applications as a building block for the construction of useful chemicals [15,16,17] and as a medium for chemical reactions [18,19].

Within this context, in the present investigation we describe the synthesis of a series of novel 1,2,3-triazoles using glycerol as the starting material. The key step involved in their preparation was the CuAAC reaction between 4-(azidomethyl)-2,2-dimethyl-1,3-dioxolane (**3**) and different terminal alkynes. The fungicide activity of the obtained 1,2,3-triazoles was assessed in vitro on the fungus *Colletotrichum gloeosporioides*, which is an important papaya crop pest. In addition, the phytotoxicity and cytotoxicity of the glycerol triazolic derivatives were evaluated on *Lactuca sativa* L. (lettuce), which is an efficient model for the investigation of toxic effects of chemical compounds [20], and the results are also discussed.

## 2. Results and Discussion

### 2.1. Synthesis

**Scheme 1:**
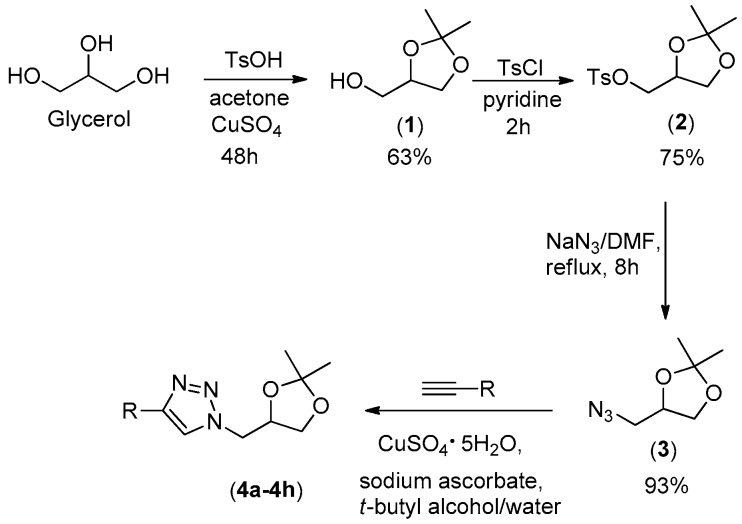

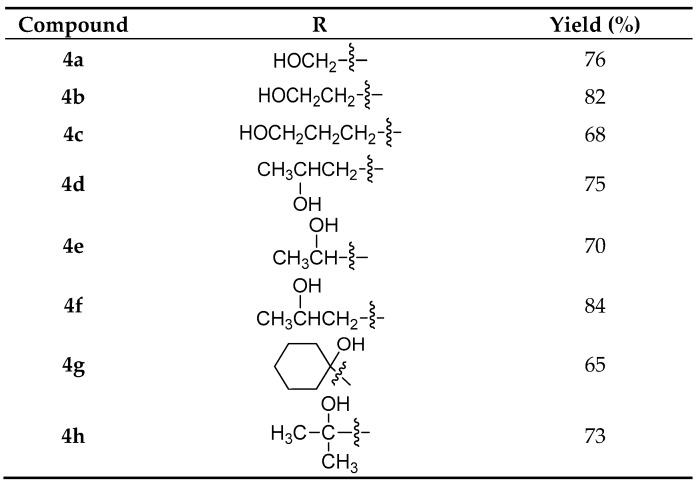
Synthesis of novel 1,2,3-triazole derivatives **4a**–**4h**. Glycerol was utilized as starting material to produce triazole derivatives in four steps. Intermediates (**1**), (**2**) and (**3**) were obtained in 63%, 75% and 93% yield, respectively. Compounds **4a**–**4h** were obtained in 68%–84% yield.

**Figure 1:**
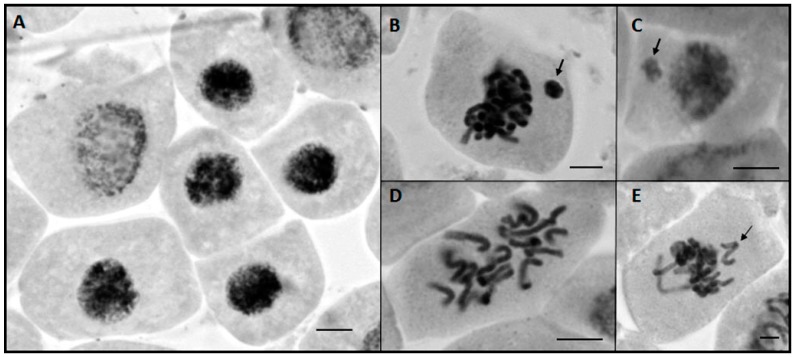
Examples of chromosomal and nuclear alterations observed in the meristematic cells of *Lactuca sativa* (lettuce) exposed to the synthesized triazoles **4a**–**4h**. (**A**) Condensed nuclei, (**B**) sticky chromosomes and micronuclei (arrow), (**C**) micronuclei (arrow), (**D**) c-metaphases, (**E**) non-oriented chromosomes in metaphase (arrow).

The new triazoles were synthesized from glycerol following the synthetic route shown in Scheme 1. The acetal **1** was obtained in 63% yield via the reaction of glycerol with acetone, catalyzed by *p*-toluenesulfonic acid. The treatment of compound **1** with *p*-toluenesulfonyl chloride afforded the ester *p*-toluenesulfonate **2** in 75% yield. The bimolecular nucleophilic substitution reaction between ester sulfonate **2** and sodium azide afforded compound **3** in 93% yield. The CuAAc reaction (click reaction) between organic azide **3** and different commercially available terminal alkynes gave rise to eight novel triazoles **4a**–**4h** in synthetically useful yields (65%−84%, Figure 1). IR and NMR (^1^H and ^13^C) spectroscopies as well as mass spectrometry analyses confirmed the structures of the synthesized compounds.

### 2.2. Biological Evaluation

The mycelial growth results (in cm) of *C. gloeosporioides* treated with the triazoles **4a**–**4h** are presented in Table 1. The triazoles presented inhibitory effects in all treatments, compared to the control. The best results were associated with compounds **4f** and **4g** which, at the highest concentration, presented 0.46 and 0.85 cm mean radial diameters, respectively.

The synthesized triazoles **4a**–**4h** also presented antisporulating activity in vitro against *C. gloeosporioides* (Table 2). The most effective compounds were **4d** and **4g** at the concentration of 1000 µg mL^−1^, presenting 2.49 and 2.99 spores mL^−1^, respectively. These compounds significantly reduced the sporulation more than 98%.

The significant antisporulating performance demonstrated by the triazoles can be explained by rapid penetration and translocation as well as toxic action on sporulation and spore germination [21]. The efficiency of 1,2,3-triazoles in reducing sporulation was demonstrated by Chen and co-workers [22]. These authors synthesized eighteen triazolic compounds and verified that they showed fungicide activity against *C. lagenarium*, inhibiting the reproduction of the phytopathogen by 61% at a concentration of 200 µg mL^−1^. In another study, Silva and colleagues [23] synthesized fifteen new 1,2,3-triazoles observed that the compounds displayed fungicide activity against *Aspergillus niger* and low cytotoxicity.

The regression models of the data obtained from the fungicide activity evaluation of triazoles **4a**–**4h** on *C. gloeosporioides* are listed in Table 3. The lowest values of ED_50_ and ED_100_ were obtained for the compounds **4f** and **4g** in relation to mycelial growth. Compounds **4g** and **4h** were the most active in terms of sporulation.

As can be seem from Table 3, the triazoles **4a**–**4h** presented lower ED_50_ and ED_100_ values for sporulation in relation to mycelial growth of *C. gloeosporioides*, meaning that such compounds have greater inhibitory action on the reproductive structures than on the vegetative growth structures. Reducing the sporulation of a phytopathogen is of great importance, since inhibition or death of these structures is directly linked to the reproduction of the species, thus affecting its propagation, reproductive cycle and resistance [24].

### 2.3. Cytotoxic and Phytotoxic Effect

The investigation of the phytotoxicity and cytotoxicy effects of triazoles **4a–4h** on *L. sativa* revealed that the percentage of germinated seeds in all treatments was higher than 98%, including the control treatments (distilled water and DCM) (Table 4). In addition, the germination speed index (GSI) of all treatments was statistically similar, not differing from the applied controls. With regard to root length (RL), variation was observed, ranging from 6.81 mm for compound **4a** to 9.91 mm for compound **4c**, at 250 µg mL^−1^ concentration, with this being the only treatment that significantly increased RL in relation to the controls. On the other hand, compounds **4a** and **4e** at 250 µg mL^−1^ and **4b** at 100 µg mL^−1^ significantly reduced RL by about 10%.

Among the macroscopic parameters evaluated in the assays to investigate the effects of chemical substances, germination appears to be the least sensitive [25]. On the other hand, root growth (RG) is directly affected by the germination delay, which is reflected in the GSI reduction as a consequence of the toxic effect of the tested compound. Hence, significant decreases in RG reflect the toxicity of the substance [26].

Evaluating the phytotoxicity of the triazolic active principles difenoconazole and tebuconazole, found in commercial fungicides, Bernardes and co-workers [27] found that the first did not affect the percentage of germinated seeds, similarly to what was observed in the present work. The triazoles tested here did not affect the GSI of lettuce seeds, whereas significant reductions in GSI were observed for difenoconazole and tebuconazole.

Root growth and initial development of the lettuce plantlet were significantly reduced only in treatments with three of the triazoles evaluated here (**4a**, 250 µg mL^−1^; **4b**, 100 µg mL^−1^; **4e**, 250 µg mL^−1^, Table 4), whereas tebuconazole and difenoconazole, in the work of Bernardes and co-workers, had significant effects on the reduction of GSI and RG at all evaluated concentrations. It is worth noting, however, that the concentrations tested in the present work are one thousand times more diluted than those utilized by Bernardes and co-workers [27], which varied from 7 to 200 g L^−1^.

The herbicide potential of the triazoles **4a**–**4h** did not become evident in the applied tests, since they did not compromise plantlet development, but the mechanism of action of triazoles presenting herbicide activity is related to photosynthesis. According to Rodrigues and Almeida [28], the commercial triazolic herbicide amicarbazone acts by inhibiting photosystem II and photosynthesis, thus preventing the fixation of CO_2_ and NADPH_2_ and affecting weed growth. Sulfentrazone is another herbicide from the triazole class and acts via inhibition of the enzyme protoporphyrinogen oxidase. This enzyme is important in the chlorophyll synthesis chain [29] which also impacts the inhibition of photosynthesis and causes death of the plant due to lack of nutrients.

Nevertheless, the mechanism of action of the triazoles **4a**–**4h** is in accordance with the observations of Borgati and co-workers [10]. In evaluating the effect of 13 triazolic derivatives containing benzyl-halogenated groups, the authors observed an RG reduction in *Allium cepa* (onion), *Cucumis sativus* (cucumber) and *L. sativa* (lettuce), comparable to 2,4-dichlorophenoxyacetic acid (2,4-D), a commercial herbicide. Furthermore, the phytotoxic action of a compound is directly related to its antiproliferative capacity and aneugenic action, hindering microtubule proliferation. In the present work, the aneugenic action was observed as discussed below.

The microscopic evaluation (Table 4) showed that, overall, the treatments with triazoles **4a** to **4e** reduced MI compared to the control treatments, whereas triazoles **4f**, **4g** and **4h** presented MI statistically similar to the control. Comparable effects were observed with regard to nuclear alterations, which comprises condensed nuclei (Figure 1A), and micronuclei (Figure 1B), where condensed nucleus was the most frequent. In this case, an increase in nuclear alterations was noticed in the treatments with triazoles **4a** to **4e**, while NA frequencies significantly identical to the controls were registered for the applied concentrations of triazoles **4f**–**4h** (Table 4).

The relationship between MI reduction and an increase in condensed nuclei, observed as the most frequent nuclear alteration in the present work, has also been reported by Palmieri and colleagues [30]. This observation is related to the fact that condensed nuclei represent cytological evidence for the occurrence of cell death [31]. Hence, an increase in the frequency of cells with condensed nucleus reflects the increase in the number of cells in death process, which will no longer undergo division, thus reducing MI.

Regarding the frequency of chromosome alterations, which may lead the cell to activate its death mechanism, a variation was observed between 0.36 for the triazole **4h** at the concentration of 50 µg L^−1^ and 1.0 for the triazole **4a** at 250 µg L^−1^ (Table 4). Significant differences were established in the treatments where the increase in the frequency of total chromosome alterations was higher than 50% that observed in the controls (Table 4).

**Figure 2:**
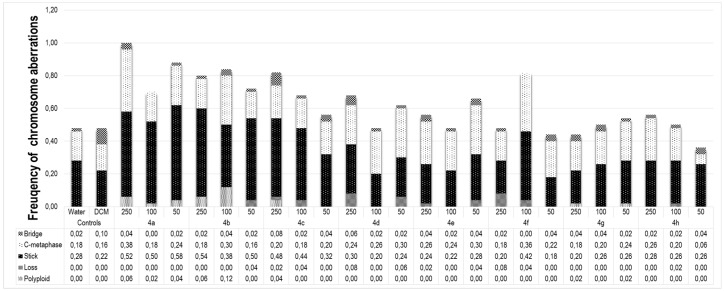
Distribution of the frequency of bridge, c-metaphase, stick, loss and polyploid observed in meristematic cells of *Lactuca sativa* using three different concentrations (50, 100 and 250 µg mL^−1^) of the triazoles **4a**–**4h**.

Distribution of the frequency of each detected alteration (bridges, c-metaphases, sticky chromosomes, non-oriented chromosomes and polyploid cells) is presented in Figure 2.

The presence of sticky chromosomes (Figure 1C) was the most frequent alteration in all evaluated treatments, followed by c-metaphases (Figure 1D and Figure 2). Among the tested treatments, only triazole **4c** at 250 µg L^−1^ presented all detected alterations simultaneously (Figure 2).

The observed chromosome alterations allow drawing conclusions concerning the mechanism of action of the tested triazoles, which is related to malfunctioning or non-polymerization of the mitotic spindle. According to Leme and Marin-Morales [32], chromosome alterations that bring about the loss or gain of chromosomes reflect aneugenic mechanisms of action of the tested compound. In addition, an aneugenic mechanism of action is attributed to the presence of c-metaphases, polyploid cells and non-oriented chromosomes in metaphase (Figure 1E) or lost in anaphase/telophase [33]. Together, these changes comprise more than 50% of all chromosome alterations observed in each treatment (Figure 2).

The presence of c-metaphases with polyploid cells is the most concrete evidence for the predominance of an aneugenic mechanism of action. Chromosomes spread across the cell and showing well-defined centromeres, which characterizes the c-metaphase (Figure 1D), arise from the absence or stabilization of the mitotic spindle [34]. In the absence of the spindle, without the possibility of segregation of the chromosomes, the cell cannot finalize the division process. Commonly, with prolongation of the compound's effect, the cell restores interphase without occurrence of chromatid segregation; as a consequence, in the subsequent cycle this cell will display twice the number of chromosomes, characterizing a polyploid cell [35,36]. The presence of non-oriented chromosomes together with the set of chromosomes on the equatorial plaque (Figure 1E), also observed in the present study as an effect of exposure to the triazoles, reinforces the aneugenic mechanism of action. These delayed chromosomes may lead to chromosome loss during the segregation process in anaphase/telophase, generating a chromosome imbalance in the daughter nuclei due to irregularities in the spindle, giving rise to daughter cells with different shapes and nuclear sizes [37].

In addition to these aneugenic alterations, the occurrence of metaphases with sticky chromosomes (Figure 1C) is also attributed to the mechanism of aneugenic action, since one of the factors responsible for their formation is related to proteins of the chromosomal protein scaffold [35]. The consequence of sticky chromosomes is abnormal chromosome segregation, which may compromise the balancing of their number in the daughter nuclei and may lead to cell death in more severe cases when the interchromatid bond [35] is very intense [33].

## 3. Materials and Methods

### 3.1. General Information

The terminal alkynes used in the click chemistry reactions were purchased from Sigma-Aldrich (St. Louis, MO, USA) and used as received. Other reagents and solvents were procured from Vetec (Rio de Janeiro, Brazil). IR spectra were obtained using a Tensor 27 spectrometer (Bruker, Karlsruhe, Germany). The samples were analyzed by attenuated total reflectance (ATR) scanning from 4000 to 500 cm^−1^. Mass spectra were recorded on a GCMS-QPPlus 2010 device (Shimadzu, Kyoto, Japan) under electron impact (70 eV) conditions of positive ion mode. ^1^H- and ^13^C-NMR spectra were recorded on a Mercury 300 instrument (Varian, Palo Alto, CA, USA) at 300 MHz and 75 MHz, respectively, using CDCl_3_ and TMS as internal standards. NMR data are presented as follows: chemical shift (*δ*) in ppm, multiplicity, the number of protons, *J* values in Hertz (Hz). Multiplicities are shown as the following abbreviations: s (singlet), brs (broad singlet), d (doublet), dd (double of a doublet), t (triplet), quart (quartet), quint (quintet), sept (septet). Melting points were obtained with PFMII equipment (Tecnopon, São Paulo, Brazil) and are not corrected. Analytical thin layer chromatography analysis was conducted on aluminum backed precoated silica gel plates using different solvent systems. TLC plates were visualized using potassium permanganate solution, phosphomolybdic acid solution and/or UV light. Flash column chromatography was performed using silica gel 60 (60−230 mesh).

### 3.2. Synthesis of (2,2-dimethyl-1,3-dioxolan-4-yl)methanol *(**1**)*

Glycerol (100 mL, 1.36 mol), acetone (100 mL, 1.36 mol), *p*-toluenesulfonic acid (0.0800 g, 0.460 mmol) and copper sulfate pentahydrate (10.0 g, 62.65 mmol) were added to a round-bottom flask. The resulting reaction mixture was stirred at room temperature for two days. After that, the mixture was filtered, yielding a bluish viscous liquid. This liquid (20 g) was purified by silica gel column chromatography eluted with hexane-ethyl acetate (3/1 *v*:*v*). Compound **1** was obtained in 63% yield as a colorless liquid. IR (cm^−1^, ${\bar{\upsilon }}_{\max}$): 3385, 2937, 1372, 1213, 1156. ^1^H-NMR (CDCl_3_) *δ*: 1.27 (s, 3H, CH_3_), 1.33 (s, 3H, CH_3_), 2.99 (brs, 1H, OH), 3.49 (dd, 1H, *J* = 11.5 Hz, *J* = 5.2 Hz), 3.58 (dd, 1H, *J* = 11.5 Hz, *J* = 4.2 Hz), 3.66 (dd, 1H, *J* = 8.2 Hz, *J* = 6.6 Hz), 3.94 (dd, 1H, *J* = 8.2, *J* = 6.6 Hz), 4.09–4.16 (m, 1H). ^13^C-NMR (CDCl_3_) *δ*: 25.0 (CH_3_), 26.4 (CH_3_), 62.8, 65.6, 76.0, 109.1. MS (*m/z*, %): 117 ([M-15]^+^, 38), 101 (22), 72 (10), 57 (25), 43 (100), 31 (12).

### 3.3. Synthesis of (2,2-dimethyl-1,3-dioxolan-4-yl)methyl 4-methylbenzenesulfonate *(**2**)*

Compound **1** (12.52 g, 98.48 mmol) and pyridine (50 mL, 640.0 mmol) were added into a round-bottom flask. The mixture was cooled in ice bath and stirred for 20 min. Next, 4-toluenesulfonyl chloride (27.00 g, 142.2 mmol) dissolved in dry dichoromethane (10 mL) was added. The resulting yellowish solution was magnetically stirred in ice bath for 2 hours. After this time, water (10 mL) was added to the reaction mixture and the phases were separated. The organic phase was washed several times with HCl (1 mol L^−1^), dried over anhydrous sodium sulfate, filtered and concentrated under reduced pressure. Compound **2** was purified by column chromatography eluted with hexane-ethyl acetate (3/1 *v*:*v*). This procedure afforded compound **2** as a colorless liquid in 75% yield. IR (cm^−1^, ${\bar{\upsilon }}_{\max}$): 2995, 2985, 1600, 1365, 1265, 1176, 978. ^1^H-NMR (CDCl_3_) *δ*: 1.30 (s, 3H, CH_3_), 1.33 (s, 3H, CH_3_), 2.45 (s, 3H, tosyl-CH_3_), 3.76 (dd, 1H, *J* = 8.8 Hz, *J* = 5.2 Hz), 3.93–4.04 (m, 3H), 4.23–4.31 (m, 1H), 7.34 (d, 2H, *J* = 8.2 Hz), 7.79 (d, 2H, *J* = 8.2 Hz). ^13^C-NMR (CDCl_3_) *δ*: 21.6 (CH_3_-tosyl), 25.1 (CH_3_), 26.6 (CH_3_), 66.1, 69.4, 72.8, 110.0, 127.9, 129.8, 132.4, 145.0. MS (*m/z*, %): 271 ([M-15]^+^, 90), 173 (10), 155 (76), 101 (86), 91 (89), 65 (24), 59 (10), 43 (100), 31(4).

### 3.4. Synthesis of 4-(azidomethyl)-2,2-dimethyl-1,3-dioxolane *(**3**)*

A round-bottom flask was charged with compound **2** (0.900 g, 3.15 mmol), NaN_3_ (1.00 g, 15.73 mmol) and DMF (3.00 mL). The resulting reaction mixture was stirred and refluxed for 8 hours. After that, the mixture was filtered yielding a yellowish liquid. The solution was extracted with ethyl acetate (3 × 30 mL). The organic phase was dried over Na_2_SO_4_, filtered and concentrated under reduced pressure. Compound **3** was obtained as a yellow liquid in 93% yield and not submitted to any further purification procedure. IR (cm^−1^, ${\bar{\upsilon }}_{\max}$): 3497, 2932, 2102, 1736, 1662, 1439, 1386, 1244, 1091, 1046, 659. ^1^H-NMR (CDCl_3_) *δ*: 1.32 (s, 3H, CH_3_), 1.42 (s, 3H, CH_3_), 3.24 (dd, 1H, *J* = 12.6 Hz, *J* = 5.4 Hz), 3.35 (dd, 1H, *J* = 12.6 Hz, *J* = 4.7 Hz), 3.72 (dd, 1H, *J* = 8.5 Hz, *J* = 5.7 Hz), 4.00 (dd, 1H, *J* = 8.5 Hz, *J* = 6.3 Hz), 4.19–4.26 (m, 1H). ^13^C-NMR (CDCl_3_) *δ*: 25.4 (CH_3_), 26.6 (CH_3_), 53.0, 66.7, 74.7, 110.2. MS (*m/z*, %): 142 ([M-15]^+^, 39), 101 (61), 83 (4), 72 (10), 59 (12), 43 (100), 31 (5). Elemental analysis: calc. for C_6_H_11_N_2_O_2_ (MW 143.16): 50.34% C, 7.74% H, 19.57% N; found 50.42% C, 7.62% H, 19.20% N.

### 3.5. General Procedure for Copper(I)-Catalyzed Azide-Alkyne Cycloaddition Reactions for the Preparation of Triazoles ***4a–4h***

The azide **3** (1.00 g, 6.40 mmol), the terminal alkyne (9.60 mmol), an aqueous solution of *tert*-butyl alcohol (6 mL of water/6 mL of *tert*-butyl alcohol), CuSO_4_∙5H_2_O aqueous solution (0.100 mol L^−1^, 1.00 mL, 0.0960 mmol), and sodium ascorbate (0.0600g, 0.288 mmol) were added to a round-bottom flask. The reaction mixture was stirred at 50 °C for 8 h. After the end of the reaction as verified by TLC analysis, distilled water (10.0 mL) was added and the aqueous layer was extracted with dichloromethane (3 × 20 mL). The organic extracts were combined and the resulting organic phase was dried over anhydrous sodium sulfate, filtered, and concentrated under reduced pressure. The residue was purified by silica gel column chromatography eluted with ethyl acetate-methanol (9:1 *v*/*v*). The described procedure afforded triazoles **4a–4h** in 65%–84% yield. The structures of compounds **4a–4h** are supported by the following data:

*(1-((2,2-Dimethyl-1,3-dioxolan-4-yl)methyl)-1H-1,2,3-triazol-4-yl)methanol* (**4a**). Yellow liquid. IR (cm^−1^, ${\bar{\upsilon }}_{\max}$): 3355, 2972, 1472, 1365, 1202, 1040, 910, 833; ^1^H-NMR (CDCl_3_) *δ*: 1.32 (s, 3H, CH_3_), 1.37 (s, 3H, CH_3_), 3.44 (s, 1H, OH), 3.72 (dd, 1H, *J* = 8.8 Hz, *J* = 5.5 Hz), 4.10 (dd, 1H, *J* = 8.8 Hz, *J* = 6.0 Hz), 4.38 (dd, 1H, *J* = 12.9 Hz, *J* = 6.0 Hz), 4.41–4.48 (m, 1H), 4.53 (dd, 1H, *J* = 12.9 Hz, *J* = 3.3 Hz), 4.75 (s, 2H), 7.68 (s, 1H). ^13^C-NMR (CDCl_3_) *δ*: 25.3 (CH_3_), 26.9 (CH_3_), 52.5, 56.3, 66.6, 74.4, 110.6, 123.6, 148.0. MS (*m/z*, %): 213 ([M^+^], 1), 198 ([M-15]^+^, 42), 155 (49), 138 (18), 113 (18), 101 (44), 83 (10), 73 (23), 57 (23), 43 (100), 31 (12). Elemental analysis: calc. for C_9_H_15_N_3_O_3_ (MW 213.23): 50.69% C, 7.01% H, 19.71% N; found 50.76% C, 6.92% H, 19.63% N.

*2-(1-((2,2-Dimethyl-1,3-dioxolan-4-yl)methyl)-1H-1,2,3-triazol-4-yl)ethanol* (**4b**). Yellow liquid. IR (cm^−1^, ${\bar{\upsilon }}_{\max}$) ${\bar{\upsilon }}_{\max}$: 3384, 2928, 1648, 1554, 1457, 1373, 1217, 1151, 1116, 1051, 968, 880, 831. ^1^H-NMR (CDCl_3_) *δ*: 1.30 (s, 3H, CH_3_), 1.35 (s, 3H, CH_3_), 2.91 (t, 2H, *J* = 6.3 Hz), 3.40 (s, 1H, OH), 3.70 (dd, 1H, *J* = 8.8 Hz, *J* = 5.5 Hz), 3.88 (t, 2H, *J* = 6.3 Hz), 4.08 (dd, 1H, *J* = 8.8 Hz, *J* = 6.0 Hz), 4.35 (dd, 1H, *J* = 12.6 Hz, *J* = 5.4 Hz), 4.39–4.45 (m, 1H), 4.48 (dd, 1H, *J* = 12.6 Hz, *J* = 3.3 Hz), 7.52 (s, 1H). ^13^C-NMR (CDCl_3_) δ: 25.4 (CH_3_), 26.9 (CH_3_), 28.9, 52.5, 61.6, 66.6, 74.3, 110.4, 123.3, 145.4. MS (*m/z*, %): 228 ([M^.+^], 2), 227 (M^+^, 2), 212 ([M-15]^+^, 35), 197 (8), 169 (65), 152 (16), 127 (15), 110 (20), 101 (25), 68 (37), 57 (62), 43 (100), 32 (50). Elemental analysis: calc. for C_10_H_17_N_3_O_3_ (MW 227.26): 52.85% C, 7.54% H, 18.49% N; found 52.76% C, 7.43% H, 18.37% N.

*3-(1-((2,2-Dimethyl-1,3-dioxolan-4-yl)methyl)-1H-1,2,3-triazol-4-yl)propan-1-ol* (**4c**). Yellow liquid. IR (cm^−1,^ ${\bar{\upsilon }}_{\max}$): 3380, 2938, 1648, 1552, 1456, 1373, 1257, 1216, 1151, 1058, 969, 880, 831. ^1^H-NMR (CDCl_3_) *δ*: 1.30 (s, 3H, CH_3_), 1.34 (s, 3H, CH_3_), 1.88 (quint, 2H, *J*_1_=7.4 Hz, *J*_2_=6.3 Hz), 2.78 (t, 2H, *J* = 7.4 Hz), 3.41 (s, 1H, OH), 3.69 (t, 2H, *J* = 6.3 Hz), 3.70 (dd, 1H, *J* = 8.7 Hz, *J* = 5.7 Hz), 4.07 (dd, 1H, *J* = 8.7 Hz, *J* = 5.7 Hz), 4.35 (dd, 1H, *J* = 12.6 Hz, *J* = 5.2 Hz), 4.38-4.45 (m, 1H), 4.48 (dd, 1H, *J* = 12.6 Hz, *J* = 3.2 Hz), 7.44 (s, 1H). ^13^C- NMR (CDCl_3_) *δ*: 22.1, 25.4 (CH_3_), 26.7 (CH_3_), 32.2, 52.4, 61.7, 66.6, 74.3, 110.4, 122.6, 147.8. MS (*m/z*, %): 242 ([M^+^], 2), 241 ([M^.+^], 4), 226 ([M-15]^+^, 31), 211 (12), 197 (9), 183 (39), 166 (11), 112 (46), 101 (28), 83 (223), 68 (25), 57 (61), 43 (100), 41 (41), 31 (18). Elemental analysis: calc. for C_11_H_19_N_3_O_3_ (MW 241.29): 54.76% C, 7.94% H, 17.41% N; found 54.78% C, 7.93% H, 17.38% N.

*1-(1-((2,2-Dimethyl-1,3-dioxolan-4-yl)methyl)-1H-1,2,3-triazol-4-yl)ethanol* (**4d**). Yellow liquid. IR (cm^−1^, ${\bar{\upsilon }}_{\max}$): 3385, 2985, 1373, 1217, 1149, 1066, 892, 830. ^1^H-NMR (CDCl_3_) *δ*: 1.33 (s, 3H, CH_3_), 1.37 (s, 3H, CH_3_), 1.58 (d, CH_3_, *J* = 6.6 Hz), 3.50 (s, 1H, OH), 3.73 (dd, 1H, *J* = 8.8 Hz, *J* = 5.4 Hz), 4.10 (dd, 1H, *J* = 8.8 Hz, *J* = 5.7 Hz), 4.39 (dd, 1H, *J* = 12.9 Hz, *J* = 5.5 Hz), 4.42–4.49 (m, 1H), 4.52 (dd, 1H, *J* = 12.9 Hz, *J* = 3.0 Hz), 5.07 (quart, 1H, *J* = 6.6 Hz), 7.61 (s, 1H). ^13^C-NMR (CDCl_3_) *δ*: 23.0, 25.1 (CH_3_), 26.6 (CH_3_), 52.2, 62.9, 66.3, 74.0, 110.1, 121.4, 152.7. MS (*m/z*, %): 228 ([M^+^], 1), 227 ([M^.+^], 1), 212 ([M-15]^+^, 36), 169 (38), 152 (16), 127 (12), 101 (36), 73 (23), 57 (29), 43 (100), 31 (8). Elemental analysis: calc. for C_10_H_17_N_3_O_3_ (MW 227.26): 52.85% C, 7.54% H, 18.49% N; found 52.81% C, 7.49% H, 18.43% N.

*1-(1-((2,2-Dimethyl-1,3-dioxolan-4-yl)methyl)-1H-1,2,3-triazol-4-yl)propan-2-ol* (**4e**). Yellow liquid. IR (cm^−1^, ${\bar{\upsilon }}_{\max}$): 3384, 2985, 1648, 1552, 1457, 1373, 1216, 1151, 1117, 1065, 1045, 968, 941, 880, 831. ^1^H-NMR (CDCl_3_) *δ*: 1.26 (d, 3H, CH_3_, *J* = 6.3 Hz), 1.33 (s, 3H, CH_3_), 1.37 (s, 3H, CH_3_), 2.72–2.92 (m, 1H), 3.46 (s, 1H, OH), 3.74 (dd, 1H, *J* = 8.8 Hz, *J* = 5.7 Hz), 4.10 (dd, 1H, *J* = 8.8 Hz, *J* = 5.8 Hz), 4.15–4.17 (m, 2H), 4.39 (dd, 1H, *J* = 12.6 Hz, *J* = 5.7 Hz), 4.42–4.49 (m, 1H), 4.52 (dd, 1H, *J* = 12.6 Hz, *J* = 3.3 Hz), 7.52 (s, 1H). ^13^C- NMR (CDCl_3_) *δ*: 23.1 (CH_3_), 25.4 (CH_3_), 26.9 (CH_3_), 35.0, 52.5, 66.6, 67.3, 74.3, 110.4, 123.4, 145.5. MS (*m/z*, %): 242 ([M^+^], 1), 226 ([M-15]^+^, 31), 197 (94), 183 (12), 166 (11), 139 (22), 115 (26), 101 (22), 83 (26), 68 (50), 57 (69), 43 (100). Elemental analysis: calc. for C_11_H_19_N_3_O_3_ (MW 241.29): 54.76% C, 7.94% H, 17.41% N; found 54.78% C, 7.91% H, 17.43% N.

*2-(1-((2,2-Dimethyl-1,3-dioxolan-4-yl)methyl)-1H-1,2,3-triazol-4-yl)butan-2-ol* (**4f**). Yellow liquid. IR (cm^−1^, ${\bar{\upsilon }}_{\max}$): 3407, 2979, 1457, 1373, 1217, 1151, 1046, 993, 919, 831. ^1^H-NMR (CDCl_3_) *δ*: 0.84 (t, 3H, *J* = 7.4 Hz, CH_3_), 1.33 (s, 3H, CH_3_), 1.35 (s, 3H, CH_3_), 1.56 (d, 3H, *J* = 3.6 Hz, CH_3_), 1.89 (sept, 2H, *J* = 7.4 Hz), 3.46 (s, 1H, OH), 3.71 (dd, 1H, *J* = 8.7 Hz, *J* = 5.5 Hz), 4.09 (dd, 1H, *J* = 8.7 Hz, *J* = 6.0 Hz), 4.39 (dd, 1H, *J* = 12.9 Hz, *J* = 5.2 Hz), 4.42–4.49 (m, 1H), 4.51 (dd, 1H, *J* = 12.9 Hz, *J* = 4.4 Hz), 7.55 (s, 1H). ^13^C-NMR (CDCl_3_) *δ*: 8.5 (CH_3_), 25.4 (CH_3_), 26.7 (CH_3_), 28.2 (CH_3_), 36.1, 52.3, 66.6, 71.4, 74.4, 110.4, 121.5, 154.7. MS (*m/z*, %): 256 ([M^+^], 1), 240 ([M-15]^+^, 25), 226 (95), 180 (9), 125 (40), 101 (20), 84 (22), 57 (45), 43 (100), 31 (9). Elemental analysis: calc. for C_12_H_21_N_3_O_3_ (MW 255.31): 56.45% C, 8.29% H, 16.46% N; found 56.38% C, 8.25% H, 16.42% N.

*1-(1-((2,2-Dimethyl-1,3-dioxolan-4-yl)methyl)-1H-1,2,3-triazol-4-yl)cyclohexanol* (**4g**). White solid. Melting point (m.p.): 85−89 ^o^C. IR (cm^−1^, ${\bar{\upsilon }}_{\max}$): 3290, 2933, 1448, 1371, 1223, 1162, 1071, 968, 898, 828. ^1^H-NMR (CDCl_3_) *δ*: 1.34 (s, 3H, CH_3_), 1.36 (s, 3H, CH_3_), 1.52–1.99 (m, 10H), 3.48 (s, 1H, OH), 3.73 (dd, 1H, *J* = 8.5 Hz, *J* = 5.4 Hz), 4.11 (dd, 1H, *J* = 8.5 Hz, *J* = 6.0 Hz), 4.38–4.55 (m, 3H), 7.58 (s, 1H). ^13^C-NMR (CDCl_3_) *δ*: 21.9, 23.1, 25.3 (CH_3_), 26.6 (CH_3_), 38.1, 39.7, 52.1, 66.4, 69.5, 72.0, 74.0, 110.1, 121.0, 152.0. MS (*m/z*, %): 281 ([M^+^], 33), 263 (32), 248 (34), 238 (18), 210 (19), 176 (12), 152 (19), 134 (39), 121 (18), 101 (27), 79 (35), 68 (33), 57 (65), 43 (100), 31 (12). Elemental analysis: calc. for C_14_H_23_N_3_O_3_ (MW 281.35): 59.77% C, 8.24% H, 14.94% N; found 59.72% C, 8.19% H, 14.89% N.

*2-(1-((2,2-Dimethyl-1,3-dioxolan-4-yl)methyl)-1H-1,2,3-triazol-4-yl)propan-2-ol* (**4h**). Yellow liquid. IR (cm^−1^, ${\bar{\upsilon }}_{\max}$): 3388, 2983, 1457, 1374, 1212, 1150, 1052, 960, 830. ^1^H-NMR (CDCl_3_) *δ*: 1.32 (s, 3H, CH_3_), 1.34 (s, 3H, CH_3_), 1.61 (s, 3H, CH_3_), 1.62 (s, 3H, CH_3_), 2.98 (s, 1H, OH), 3.72 (dd, 1H, *J* = 8.8 Hz, *J* = 5.2 Hz), 4.09 (dd, 1H, *J* = 8.8 Hz, *J* = 5.7 Hz), 4.38 (dd, 1H, *J* = 12.6 Hz, *J* = 5.2 Hz), 4.41–4.47 (m, 1H), 4.51 (dd, 1H, *J* = 12.6 Hz, *J* = 3.3 Hz), 7.57 (s, 1H). ^13^C-NMR (CDCl_3_) *δ*: 25.1 (CH_3_), 26.6 (CH_3_), 30.2 (CH_3_), 30.4 (CH_3_), 52.1, 66.3, 68.3, 73.9, 110.1, 120.6, 155.7. MS (*m/z*, %): 241 ([M^+^], 1), 226 ([M-15]^+^, 49), 208 (11), 183 (24), 166 (10), 101 (35), 94 (24), 73 (21), 57 (38), 43 (100), 31 (12). Elemental analysis: calc. for C_11_H_19_N_3_O_3_ (MW 241.29): 54.76% C, 7.94% H, 17.41% N; found 54.73% C, 7.96% H, 17.39% N.

### 3.6. Evaluation of Fungicidal Activity

To evaluate the fungicidal effect of the triazoles **4a**–**4h** on the fungal species *Colletotrichum gloeosporioides*, aqueous solutions were prepared at 1, 10, 100, 500 and 1,000 µg mL^−1^ containing 3.5% (*v*/*v*) of dimethyl sulfoxide (DMSO). The fungicide tebuconazole was used as positive control and a 3.5% (*v*/*v*) DMSO solution as negative control. The *C. gloeosporioides* isolate was obtained from wounded papaya fruit tissues. Pure cultures were incubated in PDA (potato-dextrose-agar) culture medium at 25 °C for 10 days.

Fungitoxic activity was determined based on the sensitivity of *C. gloeosporioides* mycelial growth to the applied treatments, according to methodology described by Edgington, Khew and Barron [38] and Rampersad and Teelucksingh [39], with modifications [40].

For sporulation analysis, a spore suspension was prepared for each treatment by adding distilled water (10 mL) to the Petri dishes. With the help of a Drigalsky spatula, light friction was applied on the fungal colony to release the reproductive structures of the fungus from the culture medium. The obtained mixture was filtered with the help of a glass funnel and gauze layer. The obtained suspension was homogenized and the number of conidia was determined in a Neubauer chamber (hemocytometer).

### 3.7. Evaluation of Phytotoxicity and Cytotoxicity

The phytoxic and cytotoxic effects of compounds **4a–4h** were evaluated in order on the plant model *Lactuca sativa* L. (2n = 2x = 18) [41]. The assays were performed using three different concentrations (50, 100 and 250 µg mL^−1^) of each compound in dichloromethane and distilled water were used as negative controls.

Twenty-five lettuce seeds were placed in a 9 cm diameter Petri dish containing filter paper moistened with solution from each treatment (2 mL). The experiments followed a completely randomized design (CRD), with five repetitions per treatment. The dishes were sealed with transparent plastic film to prevent evaporation and kept moist in BOD incubator at 24 ± 2 °C without light throughout the experiment period. The number of germinated seeds was evaluated from 8 to 48 h, at 8 h intervals. The macroscopic parameters assessed were germination speed index (GSI), percentage of germinated seeds (GR), root length after 48 h (RL) and aerial growth (AG) after 120 h, as previously described [42].

For microscopic analysis, ten roots from each Petri dish were collected after 48 h of exposure, fixed in an ethanol-acetic acid solution (3:1 *v*/*v*), and stored at -4 °C for at least 24 h. The slides were prepared using the squash technique and stained with 2% acetic orcein. Slides were evaluated and the parameters mitotic index (MI), chromosomal alterations (CA) and nuclear alterations (NA) frequencies were determined.

### 3.8. Statistical Analyses

For the fungicidal assays, a completely randomized design (CRD) was adopted using a 9 × 5 + 1 factorial scheme, with nine treatments (triazoles **4a**–**4h** and tebuconazole), five concentrations (1, 50, 100, 500 and 1,000 µg mL^−1^), and one additional treatment (negative control). The data on mycelial growth and sporulation sensitivity were subjected to analysis of variance. When the ANOVA was significant at a 5% probability level, the quantitative factor (concentrations) were evaluated by means of regression analysis, and the qualitative factor (triazole compounds) by the means clustering test of Scott-Knott. The values of the percentage of fungal growth inhibition (GIP) and sporulation inhibition (SIP) [21] were used to determine ED_50_ and ED_100_ (concentration of the fungicide active ingredient necessary to inhibit by 50% and 100% the mycelial growth and sporulation of the pathogen) by means of adjustments to the regression equations [39,40].

To assess phytotoxicity and cytotoxicity, the experiments were done in a CRD using a 8 × 4 + 2 factorial scheme, with eight treatments (triazoles **4a**–**4h**), three concentrations (50, 100 and 250 µg mL^−1^), and two additional treatments (water and dichoromethane). When significant, the obtained phytotoxicity and cytotoxicity data were subjected to analysis of variance and the means were compared by the Tukey test at a 5% significance level.

## 4. Conclusions

The four-step methodology presented in this work demonstrates that chemical transformations using glycerol as a starting material were effective for obtaining new triazoles in a quick and efficient way. The compounds 1-(1-((2,2-dimethyl-1,3-dioxolan-4-yl)methyl)-1*H*-1,2,3-triazol-4-yl)cyclo-hexanol (**4g**) and 2-(1-((2,2-dimethyl-1,3-dioxolan-4-yl)methyl)-1*H*-1,2,3-triazol-4-yl)propan-2-ol (**4h**) were highly efficient when compared with the commercial fungicide tebuconazole in inhibiting the sporulation of *C.* *gloeosporioides*, with ED_50_ values of 0.44 and 0.83 mg L^−1^. In addition, compounds **4g** and **4h** do not exert either phytotoxicity or cytotoxicity against the plant model *Lactuca sativa* L. Compounds **4g** and **4h** thus stand out among the other triazoles synthesized here as potential candidates for developing novel antifungal agents against *C. gloeosporioides*. Glycerol-derived 1,2,3-triazoles may thus represent a novel scaffold to be exploited aiming at the development of new active ingredients for fungus control. Work is currently in progress in our laboratories to synthesize new derivatives with increased effectiveness.

## Figures and Tables

**Table 1:** Mean values for mycelial growth (cm) of *Colletotrichum* *gloeosporioides* treated with triazoles (**4a**–**4h**).

Compounds	Concentrations (µg mL^−1^)				
	1	10	100	500	1000
**4a**	5.39 e *	5.36 d	5.01 e	4.02 c	1.74 d
**4b**	5.72 d	5.53 d	5.29 d	3.86 d	2.64 b
**4c**	5.75 d	5.73 c	5.65 c	4.88 b	2.33 c
**4d**	5.93 d	5.90 b	4.92 e	3.72 d	1.66 d
**4e**	6.15 c	6.15 b	5.27 d	4.07 c	1.05 e
**4f**	6.17 c	6.10 b	5.97 b	1.50 g	0.46 g
**4g**	6.57 b	6.19 b	5.63 c	2.86 f	0.85 f
**4h**	6.06 c	6.18 b	5.64 c	3.27 e	1.22 e
**Tebuconazole**	1.83 f	0.30 e	0.00 f	0.00 h	0.00 h
**Control**	7.14 a	7.14 a	7.14 a	7.14 a	7.14 a

* Means followed by the same letter in the column do not differ at 5% probability by the Scott-Knott test.

**Table 2:** Mean values for spores mL^−1^ of *Colletotrichum* *gloeosporioides* treated with Triazoles **4a**–**4h**.

Compounds	Concentrations (µg mL^−1^)				
	1	10	100	500	1000
**4a**	148.28 b *	145.33 b	78.78 b	37.40 b	18.61 b
**4b**	145.15 b	125.40 c	71.61 c	37.66 b	17.51 b
**4c**	128.30 c	99.06 d	45.69 f	37.04 b	19.83 b
**4d**	102.16 e	85.48 e	66.66 d	13.79 e	2.49 d
**4e**	114.46 d	74.59 g	58.13 e	28.10 c	19.00 b
**4f**	112.30 d	79.54 f	49.54 f	19.34 d	4.68 c
**4g**	86.72 f	46.18 h	30.28 h	12.26 e	2.99 d
**4h**	83.04 f	74.41 i	41.60 g	10.20 e	3.45 c
**Tebuconazole**	9.35 g	3.28 m	0.00 i	0.00 f	0.00 e
**Control**	206.70 a	206.70 a	206.70 a	206.70 a	206.70 a

* Means followed by the same letter in the column do not differ at 5% probability by the Scott- Knott test.

**Table 3:** Regression equation models obtained from fungicide evaluation of triazoles (**4a**–**4h**) and tebuconazole against *Colletotrichum* *gloeosporioides*.

Solutions	Regression Equations		ED_50_ (µg mL^−1^)		ED_100_ (µg mL^−1^)	
	MG	SP	MG	SP	MG	SP
**4a**	Y = 23,742 + 0.049x ** R^2^ = 0.98	Y = 4145 + 0.6421 logx ** R^2^ = 0.91	529.50	21.48	1537.77	1439.60
**4b**	Y = 16,937 + 0.046x ** R^2^ = 0.92	Y = 4295 + 0.608 logx * R^2^ = 0.99	715.72	14.45	1798.08	1384.10
**4c**	Y = 17,159 + 0.048x ** R^2^ = 0.90	Y = 4642 + 0.515 logx ** R^2^ = 0.91	682.63	4.97	1.72194	1.42583
**4d**	Y = 19,419 + 0.057x ** R^2^ = 0.97	Y = 4033 + 0.494 logx * R^2^ = 0.81	529.94	0.98	1396.40	1048.40
**4e**	Y = 14,677 + 0.070x ** R ^2^= 0.99	Y = 4823 + 0.463 logx ** R^2^ = 0.99	502.20	2.41	1213.07	1425.00
**4f**	Y = 19,205 + 0.078x * R^2^ = 0.88	Y = 25.6 + 20.7x − 1.2x^2^ ** R^2^ = 0.99	394.80	10.70	1035.83	1112.17
**4g**	Y = 8334 + 0.084x ** R^2^ = 0.97	Y = 5183 + 0.517 logx ** R^2^ = 0.99	496.02	0.44	1091.26	1069.37
**4h**	Y = 14,334 + 0.069x ** R^2^ = 0.98	Y = 5043 + 0.514 logx^2^ ** R^2^ = 0.95	519.76	0.83	1248.41	1098.15
**Tebuconazole**	Y = 5627 + 1.097 logx^ns^ R^2^ = 0.55	Y = 6598 + 0.640 logx^2^ ** R^2^ = 0.99	0.26	<1	35.32	13.71

MG = Mycelial growth; SP = Sporulation; ED_50_ and ED_100_ correspond to, respectively, minimum concentration necessary to inhibit 50% and 100% of mycelial growth and pathogen sporulation. Regression coefficient followed by * or ** are significant at the 5 or 1% probability, respectively.

**Table 4:** Macroscopic and microscopic parameters evaluated in *Lactuca sativa* seeds treated with the triazoles **4a**–**4h** at three concentrations and negative controls (distilled water and dichloromethane).

Compounds	Concentrations (µg mL^−1^)	G%	GSI	RG	MI%	CA%	NA%
4a	50	98.40 a *	11.13 ab	7.76 ab	6.88 c	0.88 a	0.36 c
	100	100.00 a	11.12 ab	8.08 ab	6.70 d	0.78 a	0.40 c
	250	98.40 a	11.70 ab	6.81 ab	6.78 d	1.00 b	0.36 c
4b	50	98.40 a	11.43 ab	7.22 ab	6.76 d	0.72 a	0.38 c
	100	98.40 a	10.97 ab	7.13 ab	6.26 d	0.96 b	0.38 c
	250	100.00 a	11.35 a	9.05 a	6.90 c	0.80 a	0.34 c
4c	50	100.00 a	11.27 a	8.18 a	6.94 c	0.80 a	0.30 c
	100	98.40 a	11.01 a	8.54 a	6.26 d	0.68 a	0.40 c
	250	100.00 a	10.43 a	9.91 a	7.36 c	0.82 a	0.52 d
4d	50	100.00 a	11.28 a	8.20 a	7.10 c	0.62 a	0.20 a
	100	98.40 a	10.98 a	8.77 a	7.16 c	0.64 a	0.28 c
	250	99.20 a	10.57 a	8.80 a	7.20 c	0.68 a	0.30 c
4e	50	100.00 a	11.37 a	7.37 a	7.3 c	0.66 a	0.34 c
	100	99.20 a	10.73 a	7.88 a	6.88 c	0.48 a	0.36 c
	250	99.20 a	11.46 a	6.92 a	7.20 c	0.56 a	0.16 a
4f	50	100.00 a	11.51 a	8.13 a	7.88 a	0.44 a	0.06 b
	100	97.60 a	10.77 a	8.17 a	7.76 a	0.82 a	0.20 a
	250	99.20 a	11.31 a	7.84 a	7.26 c	0.48 a	0.24 a
4g	50	100.00 a	11.05 a	7.66 a	7.9 a	0.54 a	0.20 a
	100	100.00 a	11.53 a	7.87 a	8.14 b	0.50 a	0.08 b
	250	96.80 a	10.35 a	7.95 a	7.96 a	0.44 a	0.06 b
4h	50	100.00 a	11.66 a	8.07 a	8.00 a	0.36 c	0.02 b
	100	99.20 a	11.47 a	7.95 a	8.04 a	0.50 a	0.08 b
	250	100.00 a	11.27 a	9.26 a	8.12 b	0.56 a	0.12 a
water	0	98.40 a	10.90 a	7.52 a	7.88 a	0.48 a	0.16 a
dichloromethane	0	100.00 a	11.31 b	7.48 b	8.10 b	0.48 a	0.14 a

G% = Germination; GSI = Germination speed index; RG = Root growth; MI% = Mitotic index; CA% = Chromosome aberrations; NA% = Nuclear aberrations. * Means followed by the same letter do not differ statistically by the Tukey test (*p* < 0.05).
